# Perceived barriers to seeking treatment for alcohol use disorders among the general Danish population – a cross sectional study on the role of severity of alcohol use and gender

**DOI:** 10.1186/s13690-023-01085-4

**Published:** 2023-04-23

**Authors:** Sara Wallhed Finn, Anna Mejldal, Anette Søgaard Nielsen

**Affiliations:** Unit of Clinical Alcohol Research, Institute of Clinical Research, University of Southern Denmark, J.B. Winsløws Vej 20, entrance. 220 B, Odense, 5000 Denmark

**Keywords:** Alcohol use disorders, Treatment seeking, Barrier, Gender, Denmark

## Abstract

**Background:**

A minority of all individuals with alcohol use disorders (AUD) seek treatment. Since the suffering from AUD has severe consequences for both the individual and for society, it is important to improve the understanding of barriers to seeking treatment. Most studies of barriers thus far have been conducted in the United States of America or the United Kingdom. There is a need for studies from other contexts. The overall aim is to investigate barriers to treatment seeking for AUD. The specific aims are to: 1) describe barriers to AUD treatment at different levels of alcohol use. 2) compare gender differences regarding barriers to AUD treatment.

**Methods:**

Study design: Cross-sectional. Participants: 1594 representative Danish adults from the general population aged 30–65 years. An online questionnaire was administrated by a market research company. The questionnaire covered demographic data, barriers to treatment and level of alcohol use. Analyses were performed by means of chi-2 test and logistic regression.

**Results:**

The most common barriers were related to stigma and shame: admitting to others of having a problem, being labelled, fear of the consequences and that others would find out. Participants with higher severity of alcohol use were more likely to endorse a wish to handle alcohol problems themselves and to report barriers related to treatment services. Women with high severity of alcohol use, endorsed higher level of fear of the consequences than men.

**Conclusions:**

There is an urgent need to reduce stigma around AUD. Individuals with higher severity of alcohol use report a lower willingness to seek professional treatment if a problem occurs. Especially among individuals with high severity of alcohol use there is a need to address gender specific barriers.

## Background

Alcohol use disorders (AUD) are characterized by a strong desire to drink alcohol; impaired control over alcohol intake; withdrawal symptoms when ceasing or reducing use of alcohol; increased tolerance; a great amount of time spent on or recovering from alcohol use; and continued use despite problems [1]. Globally circa 100 million individuals are estimated to suffer from AUD [2]. It is estimated that 4.2% of all Disability Adjusted Life Years (DALY) worldwide are attributable to alcohol use. Moreover, alcohol use also causes substantial harm to others [3, 4].

Even though AUD is associated with great suffering both for the affected individual and others, it has, compared to other psychiatric disorders, one of the largest gaps between the number of individuals affected and the number of individuals in treatment [5]. Estimates from 26 countries worldwide suggest only 7% of individuals with substance use disorders receive treatment [6]. There are gender differences in treatment seeking, where a larger proportion of men with AUD seek treatment at some point in their lives compared to women with AUD [7]. Another important factor associated with treatment seeking is the severity of AUD. Most individuals seeking treatment have a high severity of AUD, while the much larger group suffering from mild to moderate severity of AUD seldom seek treatment [8, 9], even though treatment seeking is associated with improved outcomes [10]. To reduce the alcohol-related harms in society it is of high importance to improve treatment coverage.

In Denmark treatment services for AUD are readily available and free of cost for the individual; still only 10–15% of the individuals with AUD seek treatment [11]. To increase the rate of treatment seeking, and decrease the treatment gap, there is a need to understand why individuals with AUD do not seek treatment. One approach is to investigate beliefs around barriers to seeking treatment in the general population.

Saunders et al. [12] have proposed a model of treatment seeking, consisting of four steps with unique barriers at each step. The first step is recognizing having an alcohol problem. The second, deciding change is needed, is followed by the third step - deciding that treatment is needed. The fourth and last step is actual seeking treatment. Barriers towards treatment seeking are divided into “person-related” and “treatment-related”. Person-related barriers include cognitive or emotional features, such as not recognizing one’s alcohol use is problematic or a feeling of shame. Treatment-related barriers include knowledge about treatment possibilities, potential scarce availability of treatment options or negative emotions towards the types of treatment offered. Person-related barriers are most common in the first two steps of treatment seeking, while the latter two steps often include a combination of person-related and treatment-related barriers.

Previous studies show that barriers to seeking AUD treatment differ between men and women [13, 14]. Women report higher barriers to seeking treatment, where higher level of stigma is one barrier, and another is less access to treatment. Barriers to treatment are also likely to vary between countries and different groups in society, due to differences in treatment systems and the social norms surrounding alcohol use and help seeking. However, the majority of studies on this topic have been conducted in the United States and United Kingdom, and many studies are also limited to including only small sample sizes [15]. Hence there is a need to broaden the perspectives, and for studies from other contexts, using larger samples.

## Method

The overall aim of this study is to investigate perceived barriers to treatment seeking for alcohol use disorders in the general population.

The specific aims are twofold:

1. to describe perceived barriers to AUD treatment at different levels of alcohol use.
2. to compare if there are gender differences regarding perceived barriers to AUD treatment.

### Study design

Cross-sectional study.

### Participants

The participants were recruited by a market research company with access to a panel consisting of adults from all regions in Denmark. Between June and October year 2020, a group of adults aged 30–65 years representative of the general Danish population in that age-category were asked to participate in an online questionnaire. The topic of the survey was not known to the participants beforehand. The proportion of participants that dropped out before completing the survey was a bit higher compared to similar surveys on other topics: 8.5% compared to normally 5–6%. In total 1594 individuals participated.

### Outcome

The outcome measures were barriers to seeking AUD treatment, where the participants were asked: “Imagine, you developed an alcohol problem. Which would be your primary barriers to seeking help?”. In total 13 different barriers were explored, and the order in which they were presented were randomized; “Having to admit to others that I have a problem”, “To be labelled (stigmatization)”, “Fear of the consequences (e.g. losing a job, not spending time with children)”, “The belief that I can handle it myself”, “That others would find out”, “The price of treatment (that it is expensive)”, “To be registered”, “That it will oblige me to change my lifestyle”, “The uncertainty about what treatment entails”, “Fear that the process will be too extensive”, “That I cannot take time off from work”, “I do not know where to seek help” and “That I do not have time”. The participants answered “yes” or “no” to each barrier.

### Measurements

The questionnaire covered demographic data on sex, age, education and having children. Alcohol use and related problems were assessed with the Alcohol Use Disorder Identification Test 10 (AUDIT 10) [16]. The total score of the AUDIT 10 was categorized into three groups according to severity of alcohol use:

**Table 1:** Categorization according to severity of alcohol use

Severity of alcohol use	Total AUDIT* score Female	Total AUDIT* score Male
Low risk alcohol use	0–6	0–8
Hazardous alcohol use	7–15	9–15
Alcohol use disorder (AUD)	16–40	16 − 40

*Alcohol Use Disorder Identification Test

### Data analyses

After describing the sample, chi-2 analyses were performed to compare differences between groups. First, differences in demographics between men and women were tested. Secondly, differences between endorsement of the different perceived barriers between severity of alcohol use were tested. Where the analysis indicated significant results (p value < 0.05), the tab_chi command was applied to calculate the adjusted residuals in order to test which groups differed [17]. Complete-case logistic regression was performed to model dichotomous outcomes. Odds ratios (OR) were calculated with 95% confidence intervals (CI). The analyses were performed in three steps. First, the crude associations between each exposure variable were calculated with sex in the model. Secondly, all analyses were adjusted for sex, age category, education, having children and severity of alcohol use grouped in the three groups described above: low risk alcohol use, hazardous alcohol use and AUD. Moreover, an interaction between sex and severity of alcohol use was included in the model. Thirdly, differences between sex and severity of alcohol use were estimated with the lincom command, which is a linear combination of parameters. All analyses were carried out using Stata MP 16.1 (StataCorp LP, College Station, TX).

## Results

Table 2 presents an overview of the demographics of the participants, presented for the total sample, and divided by gender. A slight majority were women. Half of the participants were older than 50 years of age, and the men were older compared to the women. One third of the participants had children, and almost two thirds had 12 years or longer education. 68% had low risk alcohol use, 26% of the participants had an AUDIT score indicating hazardous alcohol use, defined as score above 6 for women and above 8 for men, and 5% scored above 15 on the AUDIT, indicating a high probability of fulfilling the criteria for AUD.

**Table 2:** Demographic data presented for all participants and divided by gender

	Total	Female	Male	p-value
	N = 1594	N = 826 (51.8%)	N = 768 (48.2%)	
Age category				0.001*
30–39 years	362 (22.7%)	210 (25.2%)	152 (19.8%)	
40–49 years	438 (27.5%)	241 (29.2%)	197 (25.7%)	
50–65 years	794 (49.8%)	375 (45.4%)	419 (54.6%)	
Children				
No	1024 (64.2%)	526 (63.6%)	498 (64.8%)	0.628
Yes	570 (35.8%)	300 (36.4%)	270 (35.2%)	
Education				0.059
Up to 12 years	180 (11.3%)	89 (10.8%)	91 (11.8%)	
Vocational training	389 (24.4%)	184 (22.3%)	205 (26.7%)	
> 12 years	1021 (64.1%)	552 (66.8%)	469 (61.1%)	
Missing	4 (0.3%)	1 (0.1%)	3 (0.4%)	
AUDIT* total score				
Mean (SD)	6.0 (4.8)	5.1 (4.1)	6.9 (5.4)	0.000*
Median	5	4	5	
25th percentile	3	3	3.5	
75th percentile	12	9	14	
Low risk alcohol use a)	1091 (68.4%)	556 (67.3%)	535 (69.7%)	0.000*
Hazardous alcohol use b)	416 (26.1%)	244 (29.5%)	172 (22.4%)	
AUD c)	87 (5.5%)	26 (3.2%)	61 (7.9%)	

* Alcohol Use Disorder Identification Test

a) AUDIT score below 7 for women and 9 for men

b) AUDIT score between 7 and 15 (incl) for women and between 9 and 15 (incl) for men

c) AUDIT score higher than 15 for women and for men

In Table 3, the proportion of participants, grouped according to level of alcohol use – low risk alcohol use, hazardous alcohol use and AUD - who endorsed the different perceived barriers to AUD treatment is reported. The perceived barriers varied greatly in the size of the proportion of participants who endorsed them, from the highest “Having to admit to others that I have a problem (43.2%) to the lowest “That I do not have time” (2.9%). The five most common barriers, were “Having to admit to others that I have a problem” (43.2%); “To be labelled (stigmatization)” (34.9%); “Fear of the consequences (e.g. losing a job, not spending time with children)” (27.9%); “The belief that I can handle it myself” (22.5%) and “That others would find out” (21.9%).

**Table 3:** Reported barriers to treatment for AUD* divided by severity of alcohol use

	Total	Low risk alcohol use a)	Hazardous alcohol use b)	AUD* c)	p-value
	N = 1594	N = 1091	N = 416	N = 87	
Having to admit to others that I have a problem	688 (43.2%)	483 (44.3%)	174 (41.8%)	31 (35.6%)	0.240
To be labelled (stigmatization)	557 (34.9%)	389 (35.7%)	147 (35.3%)	21 (24.1%)	0.093
Fear of the consequences (e.g. losing a job, not spending time with children)	445 (27.9%)	331 (30.3%)	99 (23.8%)	15 (17.2%)	0.003*
The belief that I can handle it myself	358 (22.5%)	225 (20.6%)	104 (25.0%)	29 (33.3%)	0.008*
That others would find out	349 (21.9%)	231 (21.2%)	102 (24.5%)	16 (18.4%)	0.270
The price of treatment (that it is expensive)	266 (16.7%)	168 (15.4%)	84 (20.2%)	14 (16.1%)	0.082
To be registered	239 (15.0%)	156 (14.3%)	68 (16.3%)	15 (17.2%)	0.510
That it will oblige me to change my lifestyle	248 (15.6%)	137 (12.6%)	80 (19.2%)	31 (35.6%)	< 0.001*
The uncertainty about what treatment entails	179 (11.2%)	128 (11.7%)	42 (10.1%)	9 (10.3%)	0.640
Fear that the process will be too extensive	171 (10.7%)	95 (8.7%)	61 (14.7%)	15 (17.2%)	< 0.001*
That I can not take time off from work	127 (8.0%)	80 (7.3%)	43 (10.3%)	4 (4.6%)	0.077
I did not know where to seek help	74 (4.6%)	50 (4.6%)	19 (4.6%)	5 (5.7%)	0.880
That I do not have time	46 (2.9%)	24 (2.2%)	17 (4.1%)	5 (5.7%)	0.039*

* Alcohol Use Disorder

a) AUDIT (Alcohol Use Disorder Identification Test) score below 7 for women and 9 for men

b) AUDIT score between 7 and 15 (incl) for women and between 9 and 15 (incl) for men

c) AUDIT score higher than 15 for women and for men

Some differences between severity of alcohol use and reported barriers were found and are reported in Table 4.

**Table 4:** Differences in barriers to treatment for AUD* divided by severity of alcohol use

	Adjusted residual likelihood-ratio chi2(2)	Low risk alcohol use a)	Hazardous alcohol use b)	AUD* c)	p-value
		N = 1091	N = 416	N = 87	
Fear of the consequences (e.g. losing a job, not spending time with children)	12.181	3.175	-2.179	-2.283	0.002*
The belief that I can handle it myself	9.032	-2.587	1.445	2.500	0.011*
That it will oblige me to change my lifestyle	32.916	-4.868	2.404	5.313	< 0.000*
Fear that the process will be too extensive	14.374	-3.838	3.017	2.019	0.001*
That I do not have time	5.878	-2.409	1.702	1.640	0.053

* Alcohol Use Disorder

A higher proportion of participants in the low-risk alcohol use group endorsed the barrier “Fear of the consequences (e.g. losing a job, not spending time with children)” (30.6%) compared to participants with hazardous alcohol use (23.8%) or AUD (17.2%). A higher proportion of participants in the groups with higher severity of alcohol use compared to low-risk alcohol use reported: “The belief that I can handle it myself” (AUD 33.3%; hazardous alcohol use 25.0% and low risk alcohol use 20.6%). A higher proportion of participants in the groups with higher severity of alcohol use compared to low-risk alcohol use reported: “That it will oblige me to change my lifestyle” (AUD 35.6%; hazardous alcohol use 19.2% and low risk alcohol use 12.6%). A higher proportion of participants in the groups with higher severity of alcohol use compared to low-risk alcohol use reported: “Fear that the process will be too extensive” (AUD 17.2%; hazardous alcohol use 14.7% and low risk alcohol use 8.7%). A higher proportion of participants in the groups with higher severity of alcohol use compared to low-risk alcohol use reported: “That I do not have time” (AUD 5.7%; hazardous alcohol use 4.1% and low risk alcohol use 2.2%).

In Table 5, gender differences regarding the five most common barriers to treatment are presented.

**Table 5:** Odds ratios for differences between men and women in endorsement of the five most common barriers

	OR crude (95% CI) n = 1594	*p*	OR adjusted * (95% CI) n = 1590	*p*
Having to admit to others that I have a problem (men reference)	1.272 (1.043; 1.552)	0.018	1.234 (0.970; 1.571)	0.087
To be labelled (stigmatization) (men reference)	1.417 (1.151; 1.744)	0.001*	1.189 (0.925; 1.529)	0.177
Fear of the consequences (e.g. losing a job, not spending time with children) (men reference)	1.429 (1.146; 1.783)	0.002*	1.240 (0.949; 1.621)	0.114
The belief that I can handle it myself (men reference)	1.007 (0.796; 1.274)	0.953	0.946 (0.704; 1.272)	0.715
That others would find out (men reference)	1.590 (1.249; 2.025)	0.000*	1.533 (1.136; 2.069)	0.005*

*adjusted for age category, education, having children and severity of alcohol use (low risk alcohol use, hazardous alcohol use or alcohol use disorder)

For four of five of the most common perceived barriers, there were no gender differences. The difference found was that women had a 50% higher odds (1.533) compared to men of endorsing the barrier “That others would find out”.

**Fig. 1:**
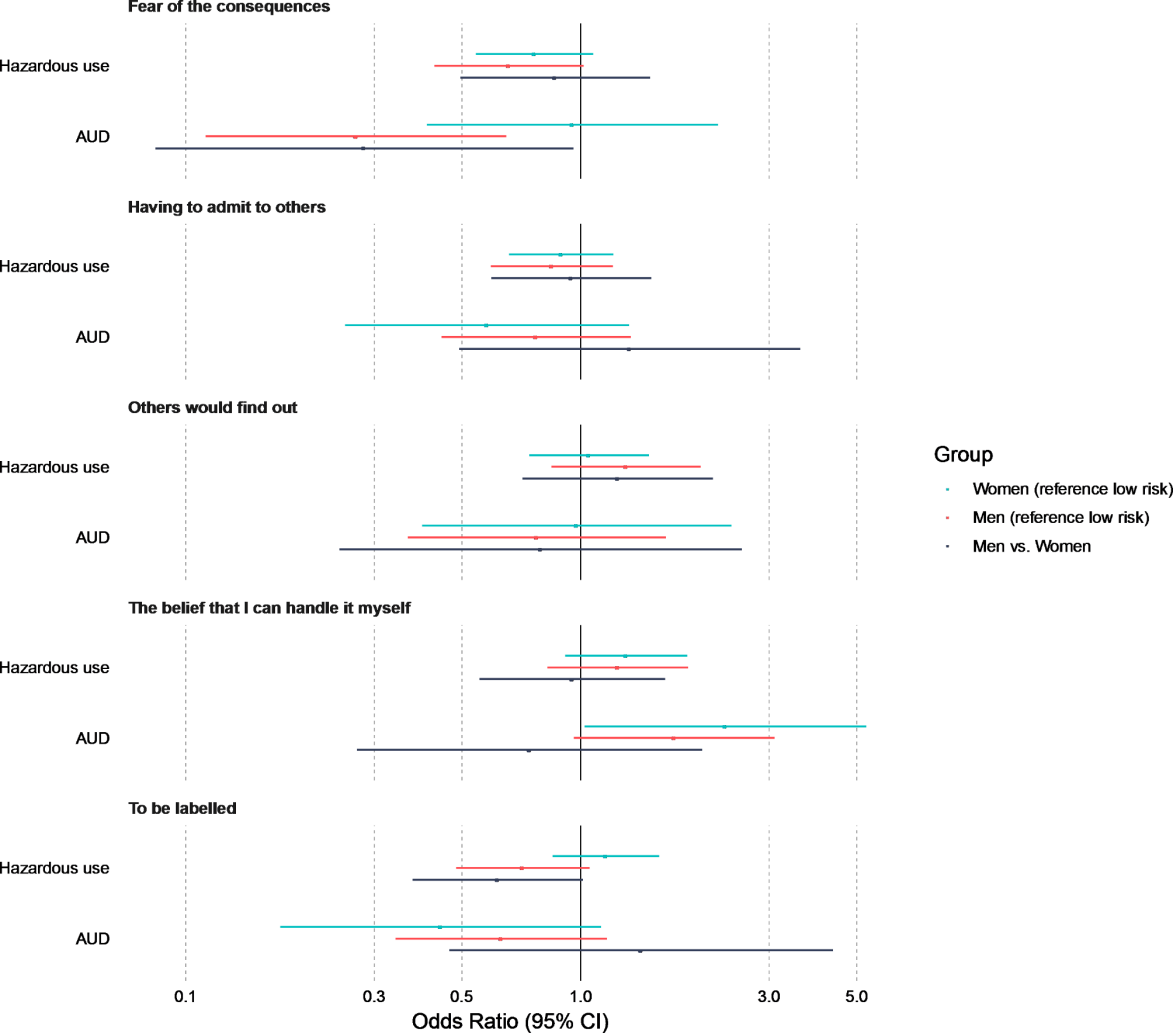
Differences in odds ratios between sex and severity of alcohol use (low risk alcohol use, hazardous alcohol use or alcohol use disorder) in endorsement of the five most common barriers

Men with AUD had a 70% lower odds (0.283) compared to women with AUD in endorsing the barrier “Fear of the consequences (e.g. losing a job, not spending time with children)”. Men with AUD had lower odds compared to men with low risk alcohol use in endorsing the barrier “Fear of the consequences (e.g. losing a job, not spending time with children)”.

Women with AUD had more than double the odds (2.326) compared to women with low risk alcohol use of endorsing the barrier “The belief that I can handle it myself”.

No other interactions between sex and severity of alcohol use for the odds of endorsing each barrier were found.

## Discussion

The aim of this cross-sectional study was twofold; firstly, to describe perceived barriers to AUD treatment at different levels of alcohol use, and secondly, to compare if there are gender differences regarding perceived barriers to AUD treatment in a general population sample. For both aims the results showed more similarities than differences between groups. First, the general implications of the results will be discussed, and thereafter the implications of the results on differences between severities of alcohol use respectively gender will be discussed.

### Barriers to treatment

The most common perceived barriers were all person-related barriers, rather than treatment-related barriers, and they were mainly associated with stigma and shame: admitting to others of having a problem, being labelled, fear of the consequences and that others would find out. These results, stemming from a larger sample size compared to many other studies, confirm previous findings from other countries and show that these also are applicable in a current Danish context. They also reiterate stigma as an important and strong barrier to seeking AUD treatment.

AUD are among the most highly stigmatized medical conditions in the Western world [18, 19]. Individuals with AUD are viewed as being more responsible for their disorder and elicit more social rejection and more negative emotions compared to other disorders. To decrease the alcohol related harm in society, there is an urgent need to reduce stigma. Educational based interventions, aiming to increase knowledge about the stigmatized group, and interventions aiming to increase social contact with the stigmatized group have shown to be effective in reducing the stigma associated with mental illness in general and SUD in specific [13, 20]. However, there is a need for high quality research in this field and studies on effects over a long-term follow up.

Another of the commonly reported barriers identified in the present study, also found in previous studies from other countries [21, 22], was the belief that “I could handle it myself.” Recovery from AUD, also in the absence of formal treatment, is common [23]. However, as treatment seeking is associated with improved outcomes [10], this raises the issue of how to increase earlier problem recognition among individuals with AUD. Problem recognition is also the first step in Saunders model of treatment seeking [12]. Screening, brief interventions and referral to treatment have been suggested as one method that is suitable to implement in health care settings in order to raise awareness of the harms associated with alcohol use, reduce alcohol use and increase treatment rates [24]. However, the efficacy of the component referral to treatment for AUD has been questioned [25], and there is a need for new approaches to narrow the treatment gap. One approach for future studies is to investigate the role of different messages around AUD and treatment seeking. Traditionally, AUD have often been described in dichotomized categories – either someone fulfill the diagnostic criteria for the diagnoses, or not. In the Diagnostic and Statistical Manual of Mental Disorders version 5 (DSM-5), severities of AUD was introduced, meaning that fulfilling the diagnosis is a matter of continuum rather than a dichotomy [26]. One approach for future studies could be to increase the use of messages including continuum beliefs around AUD and treatment seeking for AUD. The possible benefits of this are twofold, firstly the use of continuum beliefs, compared to the traditional binary belief model, has been found to reduce stigma for psychiatric disorders [27]. Secondly, there is also evidence that the use of a continuum beliefs model of AUD can increase problem recognition, which in turn can improve treatment seeking [28].

### Severity of alcohol use

There were two barriers in the personal category that were more often endorsed among those with higher severity of alcohol use compared to lower use: “The belief that I can handle it myself” and “That it will oblige me to change my lifestyle” and there were two treatment-related barriers: “Fear that the process will be too extensive” and “That I do not have time”. Saunders model [12] suggests that the latter two steps of treatment seeking often include a combination of person-related and treatment-related barriers, and it is thus reasonable to expect a combination of types of barriers among those with higher severity of alcohol use, as those individuals are more likely to be further along in the treatment seeking process compared to individuals with lower severity of alcohol use. A previous European study have suggested that not perceiving a need for treatment (if a problem should occur) is more common at lower problem severity compared to higher problem severity [29]. The results from this study show the opposite. This could be due to differences in recruitment of participants, since the previous study was performed in a primary care setting [29], while the present is based on a general population sample. However, a recent qualitative study amongst AUD-treatment-naïve individuals suffering from severe AUD, recruited at hospitals, show that fear of stigma, or lack of knowledge about treatment, may not be major reasons for not seeking treatment, despite heavy drinking. Rather, fear of losing autonomy and the perception that treatment is not a relevant option for oneself were expressed [30]. The informants described that heavy alcohol use had become a natural part of life, that they felt in control, despite the high consumption, and that they expressed confidence in being able to change by themselves, if needed. They simply did not regard themselves as individuals, who sought professional help. This personal barrier for treatment seeking may, obviously, be more pronounced among heavy drinkers, simply because alcohol has become a larger part of daily life in the heavy drinking group, compared to non-heavy drinkers.

An understudied but important area in order to reduce the treatment gap is perceptions of treatment and treatment preferences [31]. How people view treatment plays an important role in the third step in Saunders model [12] – the decision to seek treatment. The two treatment related barriers highlight the need to increase the knowledge about AUD treatment, as treatment does not need to be extensive or time consuming [32]. However, little attention has thus far been given to strategies on how to attract people with AUD to seek treatment. One approach could be the use of concepts from direct-to-customer (DTC) marketing [33]. The focus in DTC is on increasing the customers’, in this case individuals with AUD, awareness of AUD and evidence-based interventions. One possible method is via national mass media campaigns [34]. However, DTC needs careful considerations, as reservations over using this in health care services have also been raised [35].

In addition to increasing the knowledge about AUD treatment, there is also a need to focus on the content of AUD treatment to make it more engaging across different severities of AUD. For example, patient centered treatment goals – abstinence or reduced alcohol use [31, 36, 37] could be offered. Furthermore, to offer a wider range of treatment options: via the Internet [38] or a blend of the Internet and face-to-face treatment [39], both of which also can be less time consuming and easier to access compared to traditional treatment. Internet interventions have also been shown to attract a larger proportion of women compared to traditional treatment options [38].

### Gender differences

Our study suggests that in Denmark there are few differences between men and women in the perception of barriers to seeking AUD treatment. The gender differences found in this study were person-related, rather than treatment-related. This is in contrast to previous studies, where the reported barriers were rather a mix of person-related and treatment-related, and where treatment-related barriers were lack of knowledge about treatment, concerns about the treatment content, costs and concerns about childcare [40, 41]. However, the endorsement of treatment-related barriers was generally lower, and therefore potential gender differences may go undetected.

A novel finding in our study, not previously reported in the literature, was that the gender differences varied with severity of alcohol use. In the group of participants with AUD, women were more likely than men with AUD to note “fear of the consequences” as a barrier. Several different examples of consequences were given: “e.g. losing a job, not spending time with children”, which unfortunately does not make it possible to disentangle which consequences are the most significant.

There has been calls for more attention to address the specific barriers to seeking AUD treatment especially for pregnant women and women with child care responsibilities [41]. This study did not address the specific barriers that these groups can face. Moreover, women with AUD face other challenges compared to men with AUD, one being higher levels of experience of psychological, physical and sexual abuse, which has been suggested to lead to different treatment needs and possibly different needs in the treatment seeking process [15]. However, the role of gender needs further attention, as there is also evidence that men with co-occurring diagnoses report higher levels of unmet need of treatment compared to women [42].

### Strengths and limitations

A strength is the large sample size, which allowed for analyses on severity of alcohol use, and also taking education and having children into consideration in the analyses. Another strength is that the data was collected in a context outside of the USA and the UK, thus giving a wider perspective on barriers to treatment seeking.

A limitation is that there is no data on previous treatment seeking or motivation to seek treatment among the participants, which presumably are variables of importance for the outcome.

Another limitation is that the study only gives a broad picture of important variables, and that there is a lack of data on subgroups that need attention in future studies. One example was that sex was only collected as binary variable of biological sex: men or women, not including a gender perspective about the socially constructed sex. Nor does the data include certain variables that intersect with gender and that also plays a role in treatment seeking, as ethnicity or sexuality [43]. Future studies should also include a larger age span among the participants, as there are indications that age is an important factor for treatment seeking, where younger and older are less likely to perceive a need for AUD treatment [44]. Moreover, psychiatric and somatic co-morbidities are highly relevant factors associated with treatment seeking [43], but their relation to treatment barriers have received little attention. Finally, even if this study in comparison to many previous studies, have a large sample size, the group of individuals with AUD was quite small, which contributes to uncertain estimates.

## Conclusion

To lower the barriers and increase treatment seeking for AUD, there is an urgent need to reduce the stigma associated with AUD. Individuals with severe alcohol use compared to low alcohol use report a lower willingness to seek professional treatment if a problem occurs; in particular among women. Especially among individuals with high severity of alcohol use there is a need to address gender specific barriers.

## Data Availability

The data underlying this article cannot be shared publicly due to the privacy of individuals that participated in the study. The data will be shared on reasonable request to the corresponding author.

## Abbreviations

AUD: Alcohol Use Disorders
AUDIT 10: Alcohol Use Disorder Identification Test 10
CI: Confidence interval
DSM-5: Diagnostic and Statistical Manual of Mental Disorders version 5
DTC: Direct-To-Customer
OR: Odds ratio
